# The effects of induced optical blur on visual search performance and training

**DOI:** 10.1177/17470218211050280

**Published:** 2021-10-13

**Authors:** Azuwan Musa, Alison R Lane, Amanda Ellison

**Affiliations:** 1 Department of Psychology, Durham University, Durham, UK; 2 Department of Ophthalmology, International Islamic University Malaysia, Selangor, Malaysia

**Keywords:** Feature search, optical blur, perceptual learning, visual acuity

## Abstract

Visual search is a task often used in the rehabilitation of patients with cortical and non-cortical visual pathologies such as visual field loss. Reduced visual acuity is often comorbid with these disorders, and it remains poorly defined how low visual acuity may affect a patient’s ability to recover visual function through visual search training. The two experiments reported here investigated whether induced blurring of vision (from 6/15 to 6/60) in a neurotypical population differentially affected various types of feature search tasks, whether there is a minimal acceptable level of visual acuity required for normal search performance, and whether these factors affected the degree to which participants could improve with training. From the results, it can be seen that reducing visual acuity did reduce search speed, but only for tasks where the target was defined by shape or size (not colour), and only when acuity was worse than 6/15. Furthermore, searching behaviour was seen to improve with training in all three feature search tasks, irrespective of the degree of blurring that was induced. The improvement also generalised to a non-trained search task, indicating that an enhanced search strategy had been developed. These findings have important implications for the use of visual search as a rehabilitation aid for partial visual loss, indicating that individuals with even severe comorbid blurring should still be able to benefit from such training.

## Introduction

The visual field is the area that can be seen when the eyes are fixating, and a visual field defect is any area of blindness within this that can result from damage to any part of the primary visual pathway. The primary cause is glaucoma (Ramrattan et al., 2001), although there are other ocular pathologies such as macular degeneration. The area of blindness is restricted to one-half of the visual field in homonymous visual field defects, which are the consequence of damage to most typically primary visual cortex, followed by optic radiations, the optic tract, and lateral geniculate nucleus (Papageorgiou & Tsironi-Malizou, 2017). Regardless of cause, visual field defects can result in significant disability and increase the chance of falling (Ramrattan et al., 2001) and collisions (Fuhr et al., 2007; McGwin et al., 2016; Papageorgiou et al., 2012). This is because the restricted field of view hinders many everyday tasks that require efficient searching, including safe navigation, and finding items such as when shopping.

Visual search tasks involve the shifting of attention and scanning of an array to decide upon, typically, the presence or absence of a target item (Treisman & Gelade, 1980). Search tasks have been proposed as a behavioural treatment for various visual field conditions including homonymous visual field defects (e.g., Lane et al., 2010), and age-related macular degeneration, glaucoma, and retinitis pigmentosa, for example (Kuyk et al., 2010; Liu et al., 2007). The aim with such training is to use visual search to encourage patients to develop more efficient searching eye-movement strategies, which are known to be deficient across these conditions (e.g., Meienberg et al., 1981; Smith et al., 2012; Van der Stigchel et al., 2013; Zihl, 1995).

However, blurring of vision (i.e., reduced visual acuity, [VA]) is a common comorbid visual problem for many such patients (Naidoo et al., 2014). Despite the availability of optical aids such spectacles and contact lenses to treat blurring of vision, many patients remain unable to achieve satisfactory VA (Oduntan, 2005; Pascolini & Mariotti, 2012). Therefore, it is important to understand the extent to which blur affects searching behaviour, and whether this blurring is a factor that affects a participant’s ability to engage successfully with visual search training. Knowing the level of VA that is essential to give a maximum training effect could enable therapists to provide tailored vision rehabilitation; patients with an acceptable level of blurred vision could be provided with search training as it stands, whereas perhaps alternatives need to be sought in cases where the blur makes training less effective.

Studies on the association between visual search and VA have been conducted in children from as young as 4 years old (Huurneman & Boonstra, 2014; Huurneman et al., 2014; Tadin et al., 2012), and in adults up to 80 years old with eye disorders such as amblyopia and retinal diseases (Dougherty et al., 2009; Fuhr et al., 2007; Kuyk et al., 2005; Liu et al., 2007; Satgunam et al., 2012). It has been demonstrated that blurred vision has an adverse impact on visual searching in patients with moderate to profound visual impairment (Kuyk et al., 2005; Senger et al., 2017); patients showed prolonged search time and increased amplitude of eye movements which were directly proportional to their VA. However, at present no clear recommendation has been published about the minimum level of VA that could still allow the execution of efficient visual search. Therefore, Experiment 1 presented here aimed to systematically quantify how different degrees of blurred vision affect visual search performance.

With regard to visual search training, Liu et al. (2007) explored the effect of training on patients with severe to profound vision loss due to retinal diseases like age-related macular degeneration and retinitis pigmentosa (<6/60 [able to see items at 6 m that can be normally seen at 60 m] best corrected VA, and/or <20° visual field). The study demonstrated that training could significantly improve visual search speed in severely visually impaired participants, and the gains were persistent for at least 1 month after training ended. Furthermore, the efficiency of the training was comparable between those with visual impairment and healthy age-matched controls demonstrating that poor vision does not prevent participants from being able to acquire improvements in search behaviour. However, the study did not include participants with minimal or moderate vision loss and, furthermore, the stimuli used were high contrast resulting in an easy to perform task. Thus, as the authors themselves noted, it would be interesting to observe performance on more difficult search tasks to see how this affects behaviour, as well as whether improvements generalise. Previous studies with normally sighted participants have found contradictory findings with respect to whether visual search practice transfers across stimuli (Ahissar & Hochstein, 1993, 1996; Ellison & Walsh, 1998; Sireteanu & Rettenbach, 1995). Therefore, the aim of the second experiment presented here was to quantify the conditions under which visual search training can have a positive effect, examining differing levels of VA and different tasks, as well as transfer.

The experiments reported here utilise feature search tasks, where the target differs to the distractors according to one characteristic, as these have been trialled for the rehabilitation of various visual field defects. For instance, the training used by Liu et al. (2007) and Kuyk et al. (2010) for patients with ocular pathology visual impairments involved a size-based feature search task where participants had to search for a square that was twice as large as the other distractors, and decide whether it was present or absent. Although one of the tasks used here is a size-based task, two other feature searches are also used: colour and shape search. The three tasks are all used as a part of Durham Reading and Exploration (DREX), a visual search training developed for people with homonymous visual field defects (www.durham.ac.uk/drex/). As with the Liu et al. (2007) and Kuyk et al. (2010) studies, participants in the second experiment reported here completed the training across five daily sessions. Although in the current study participants did complete more trials per day (600 as opposed to 405), this is fewer trials per task type (200). The number of trials per task had to be balanced with the overall training time to ensure that participants did not become too fatigued.

## Experiment 1

### Method

#### Participants

A total of 80 volunteers (16 males, 64 females) aged between 18 and 52 years (mean age = 21.35 years; *SD* = 4.84) were recruited from Durham University, and all participants provided informed consent in accordance with the Declaration of Helsinki. All participants were either emmetropes (presenting vision of 6/6) or had corrected-to-normal vision (6/6 or better when tested). None of the participants reported having any history of progressive systemic or ocular pathology, or any cognitive dysfunction. Participants were asked about their knowledge about their current spectacle prescription and/or VA if applicable. Overall, 40 participants did not wear any refractive correction, while 40 participants wore spectacles/contact lenses.

#### Design

In this mixed design study, participants were randomly assigned to one of four optical blur conditions: no blur (VA ⩾ 6/6), mild blur (VA = 6/15), moderate blur (VA = 6/30), and severe blur (VA = 6/60). Each participant completed three different visual search tasks with their vision blurred according to their assigned group. The primary outcome measure was the mean response time (RT) recorded for each task. Ethical approval was obtained from the Psychology department ethics committee at Durham University.

#### Stimuli and procedure

##### Vision testing and manipulation

Near-distance VA was measured using an ETDRS 2000 series chart at 40 cm. Uncorrected vision testing (e.g., without glasses or contact lenses) was completed monocularly by all participants and VA was recorded as 6-m Snellen equivalent. Optical lenses where then used (if necessary) to adjust the participant’s VA so that it was in accordance with the blur condition to which they had been assigned. In all instances, participants started with their uncorrected vision and wore a trial frame in which optical lenses were then placed. In cases where participants had emmetropia, a high diopter power of plus lens was used initially (blurring up to 6/60) to avoid participants memorising the chart letters in the subsequent acuity lines. The diopter power was then reduced using an estimated method until the desired VA level was achieved. The eye was blurred monocularly so that both eyes had the same level of induced VA. In cases with participants with ametropia (unaided <6/6), if the unaided VA was the same as their assigned group then no VA manipulation was done and blank lenses were inserted into the frame. If required, their unaided VA was further blurred or partially corrected using plus or minus lenses until the desired VA was achieved for the group to which they were allocated.

##### Visual search tasks

Participants were asked to complete six blocks of trials: two blocks for each of three search tasks (colour, size, and shape). The order of the tasks was counterbalanced across participants and testing was done under normal room illumination. While completing the tests, participants placed their head on a chin rest to minimise head movements and maintain the test distance of 57.5 cm. Participants were instructed to perform all tests as accurately and as quickly as possible.

**Figure 1.**
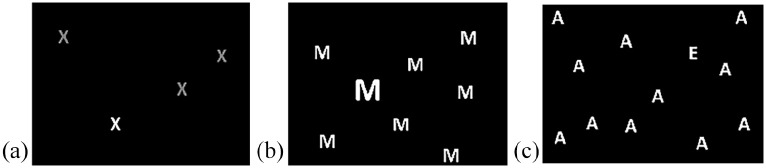
Diagrams illustrating three examples of a visual array used in the colour, size, and shape tasks (not to scale; images in greyscale): (a) the target was a letter in a different colour (e.g., white) to the distractors (grey) in a 4-item display; (b) the target was letter “M” in large size among small distractors in an 8-item display; (c) the target was letter “E” among different shape distractors in a 12-item display.

E-Prime 2.0 (Psychology Software Tools, Inc., Pittsburgh, PA) was used to create the visual search tasks, which were displayed on a 16-inch colour monitor such that the display subtended 32.5° horizontally and 24.5° vertically. The tasks were feature visual search tasks, where the target was defined by only colour, size, or shape; participants were not looking for a predefined target (e.g., the letter “M”) but rather the presence of an odd one out (see Figure 1 for examples). All items were presented on a black background, and consisted of four possible letters (E, A, X, and M) in four possible colours (cyan, magenta, red, and yellow), the combination of which was randomised. 24-point font was used for all of the items in the colour and shape tasks, and in the size tasks there was always a 10-point difference between the distractors (10–16 point) and the target (20–26 point). The items in the array were always non-overlapping and the location was random, and all distractor items were identical on any given trial. The number of items (Set-size) in each search array was 4, 8, or 12, and there were an equal number of trials for each Set-size. Half of the trials were target-present trials, and the other half were target-absent trials. The tasks consisted of 240 trials each, which were divided equally into two blocks. Each trial began with a central fixation point (white cross), which was presented for 500 ms. The arrays followed immediately and were presented for 5,000 ms, or until the participant made a response. Participants were required to press 1 to indicate the presence of the target, and 2 if they thought it was absent.

#### Statistical analyses

Analyses concentrated on the mean RT for correct target-present trials, with data from trials where the response was incorrect and outliers (*SD* values beyond calculated upper and lower quartile boundaries) removed. A (3 × 3) × 4 mixed-model ANOVA was conducted to investigate the interaction between Task (colour, size, and shape), Set-size (4, 8, and 12 items), and Group (no blur, mild blur, moderate blur, and severe blur). The sphericity of all repeated measures effects was tested using Mauchly’s test; the data were normal unless otherwise stated, and the Greenhouse–Geisser adjustment was used as required. Post hoc Bonferroni pairwise comparisons were performed when necessary to explore interactions.

### Results

The mean accuracy was above 91% in all conditions and there were no significant differences between conditions (*p* ⩾ .122). With respect to RT, the 3 (Task: colour, size, and shape) × 3 (Set-size: 4, 8, and 12) × 4 (Group: no blur, mild blur, moderate blur, and severe blur) mixed-model ANOVA revealed a statistically significant interaction between Task, Set-size, and Group, *F*(12, 304) = 2.01, *p* = .023. The analysis was therefore broken down to investigate each task.

#### Colour search task

**Figure 2.**
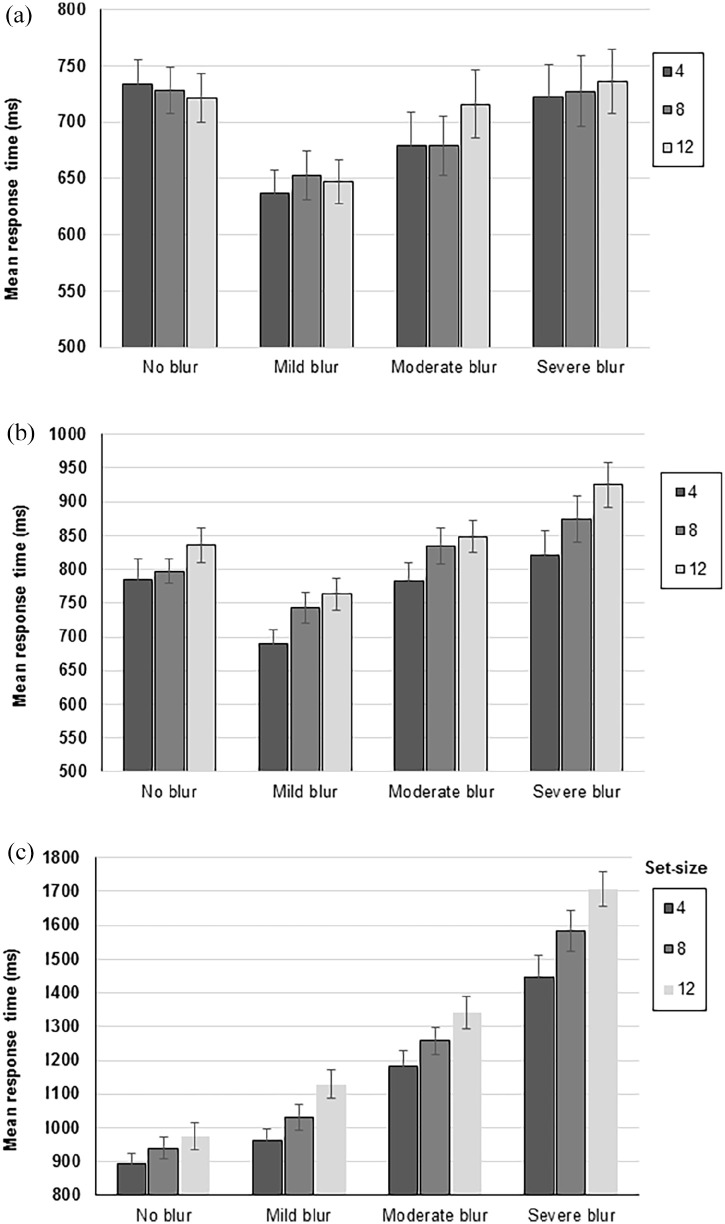
Bar charts to illustrate the mean response time (in milliseconds) for the different Set-sizes (4, 8, and 12) across four different Groups (no blur, low blur, moderate blur, and severe blur) in the (a) colour search task, (b) size search task, and (c) shape search task. The error bars represent the standard error of the mean.

The 3 (Set-size: 4, 8, and 12) × 4 (Group: no blur, mild blur, moderate blur, and severe blur) mixed-model ANOVA revealed no significant effect of Set-size, *F*(2, 152) = 1.52, *p* = .222. Search rate slopes can be calculated using the following formula (*y*2 − *y*1 / *x*2 − *x*1), and the mean search rate slope for the colour task was 1.51 ms/item (see Table 1 for the search rate slope of each Group). There was also a non-significant effect of Group, *F*(3, 76) = 2.68, *p* = .053, and no significant interaction between Set-size and Group, *F*(6, 152) = 1.54, *p* = .170; see Figure 2a.

**Table 1.** Table showing the mean search slope rate (ms/item) for each Task (colour, size, shape), and for each of the Groups (no blur, mild blur, moderate blur, severe blur).

	Colour	Size	Shape
*No blur*	−1.54	6.31	10.37
*Mild blur*	1.31	9.34	20.72
*Moderate blur*	4.61	8.01	20.02
*Severe blur*	1.65	12.87	32.64

#### Size search task

The 3 (Set-size: 4, 8, and 12) × 4 (Group: no blur, mild blur, moderate blur, and severe blur) mixed-model ANOVA revealed a main effect of Set-size, *F*(2, 152) = 54.86, *p* < .001; as the Set-size increased, the mean RT increased (Figure 2b), and the mean search rate slope was 9.13 ms/item (see Table 1 for the search rate slope of each Group). The main effect of Group was significant, *F*(3, 76) = 5.02, *p* = .003, such that the mean RT increased as the severity of blur increased. There was a non-significant interaction between Set-size and Group, *F*(6, 152) = 2.00, *p* = .069.

#### Shape search task

The 3 (Set-size: 4 items, 8 items and 12 items) × 4 (Group: no blur, mild blur, moderate blur and severe blur) mixed-model ANOVA revealed a significant effect of Set-size, *F*(2, 152) = 62.11, *p* < .001; as Set-size increased so too did the mean RT (Figure 2c). The mean search rate slope for the shape task was 20.94 ms/item. There was also a significant effect of Group, *F*(3, 76) = 38.72, *p* < .001; participants from the no blur group performed significantly faster than those from the moderate blur or severe blur groups (*p* ⩽ .007), however no significant differences in the mean search time were found in other blur condition comparisons (*p* ⩾ .647).

A significant interaction between Set-size and Group, *F*(6, 152) = 3.04, *p* = .008, was also observed. To investigate this interaction further, mean search rate slopes for each level of blur were calculated (see Table 1). These were compared using a single-factor between-subject ANOVA, which revealed that there was a significant effect of Group, *F*(3, 79) = 4.567, *p* = .005; participants from the no blur group had a significantly shallower search slope compared with the severe blur group (*p* = .003). Other group comparisons were not significantly different (*p* ⩾ .239).

### Interim discussion

The results of the first experiment show that blurred vision significantly affects visual search performance, however, this is task dependent, with the colour search task being relatively unaffected by the extent of the blur. In other words, participants in the blurring groups were equally fast on the colour search task as the no blurred controls across all Set-sizes, but become slow and inefficient on the size and shape search tasks with increasing blur. However, there is an exception for those with 6/15 acuity; they can perform very well in all visual search tasks showing that the visual search results obtained from people with minimal blurred vision can still be considered as an efficient search.

In sum, this experiment revealed that visual search speed reduces in size and shape search tasks as the severity of blurred vision increases, indicating a serious disability which could limit the execution of most activities that require efficient visual search like navigation and finding objects. Therefore, the next experiment will investigate if this impaired visual search due to blurred vision can be improved via search training.

The results of this experiment showed that there was no significant difference in mean RT between mild and moderate blur groups in all search tasks. Therefore, in the subsequent experiment a low blur (6/24) group was used, which was chosen as the mid-point VA between 6/15 (mild blur) and 6/30 (moderate blur).

## Experiment 2

### Method

#### Participants

Thirty volunteers (12 males, 18 females) aged between 18 and 35 years (mean age = 23.5 years; *SD* = 0.90) were recruited from Durham University. The inclusion criteria were the same as Experiment 1. In total, 15 participants did not wear any refractive correction, while 15 participants wore spectacles/contact lenses. All participants provided informed consent to participate in the study in accordance with the Declaration of Helsinki.

#### Design

In this mixed design study, participants completed vision testing and pre-training assessments before performing five sessions of search training, and then repeating the same assessments in a post-training session. The primary outcome measures were the mean RT and accuracy of the colour, size, and shape feature search tasks, as well as a non-trained find-the-number search task. Ethical approval was obtained from the Psychology department ethics committee at Durham University.

#### Stimuli

##### Vision testing and manipulation

The methods used for testing near-vision and allocating participants into the experimental groups were identical with Experiment 1, except that this experiment only included three experimental groups: no blur (6/6), low blur (6/24), and severe blur (6/60).

##### Pre- and post-training assessments

**Figure 3.**
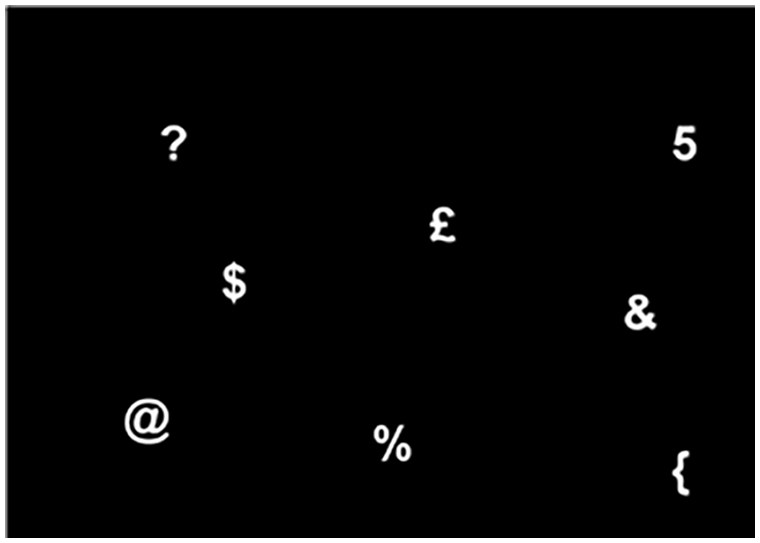
Diagram illustrating an example of a visual array used in the “find-the-number” search task (not to scale). The target used in this example was number 5.

1. Colour, size, and shape search tasks: these three tasks were the same as used in Experiment 1.
2. Find-the-number search task: the task was programmed using E-Prime 2.0 (Psychology Software Tools, Inc., Pittsburgh, PA). Participants had to scan an array of randomly displayed, non-overlapping items for a target (a number between 1 and 9). The distractors were non-numerical symbols (e.g., #, @, %, }, $, £, ?), and on half of the trials there were three distractors, and the other half of trials contained seven distractors. The distractors and target were 24-point size, white, and presented on a black background (see Figure 3), with the array displayed on a 15.6-in. laptop monitor. Once participants had identified the target they had to indicate their response as quickly as possible by pressing the spacebar followed by the corresponding number key indicating the number seen. The task consisted of 8 practice and 40 test trials. Only trials in which the correct response was provided were used for the mean RT calculation.

##### Training

The search training consisted of three visual search tasks where the target item and distribution of trials were the same as the ones used in the colour, size, and shape search tasks. However, the number of items (Set-size) in each array was 10 (including one target), and it remained constant throughout the training. The training was divided into five sessions and each session consisted of two blocks of colour, size, and shape tasks. Every block comprised 100 trials, thus making 3,000 trials in total across the sessions.

#### Procedure

After testing VA, participants were asked to complete all four assessment tasks under the VA condition they had been allocated to. A break between tasks was given if required. Participants then completed five sessions of search training: one session per day across the course of a week, with each session lasting approximately 30 min. They then repeated the assessment tasks in one final session. The assessments and training were done under normal room illumination and the chin rest was used throughout the assessments and training sessions to maintain the head position and testing distance. Participants were instructed to perform the tasks as accurately and as quickly as possible. A feedback screen summarising their performance and accuracy was displayed at the end of each assessment or training block.

#### Statistical analyses

Analyses for the assessment and training feature search tasks were restricted to correct target-present responses such that incorrect responses and outliers (*SD* values beyond calculated upper and lower boundaries) were removed. Paired-samples *t*-tests were performed on the training data, comparing performance at Session 1 with Session 5 for each blur condition separately. A (3 × 2) × 3 mixed-model ANOVA was conducted for each of the colour, size, and shape search tasks, with the factors Set-size (4 items, 8 items and 12 items), Session (pre- and post-training), and Group (no blur, low blur and severe blur). A 2 × 3 mixed-model ANOVA was conducted for the find-the-number search task with the factors Session (pre- and post-training) and Group (no blur, low blur, and severe blur). The sphericity of all repeated measures effects was tested using Mauchly’s test; the data were normal unless otherwise stated, and the Greenhouse–Geisser adjustment was used as required. In addition, post hoc Bonferroni pairwise comparisons were performed if necessary.

### Results

#### Training

**Figure 4.**
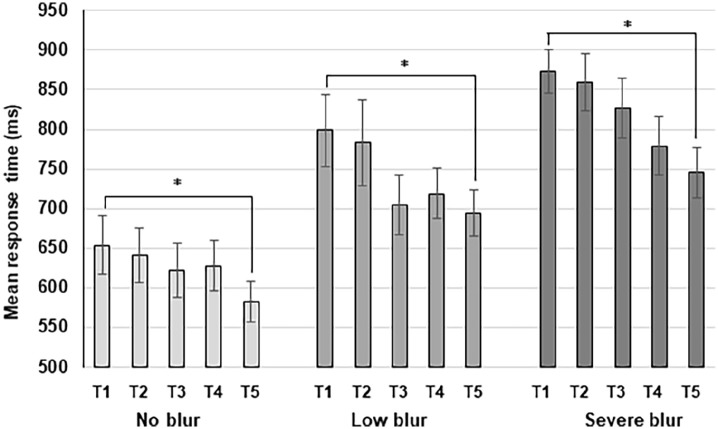
Graph to illustrate the mean response time (in milliseconds) for each group, for each training session. The error bars represent the standard error of the mean and “*” represents a significant difference.

The training data were collapsed across the three training tasks, and the mean RT (target-present condition) for each training session was calculated for each blur group (see Figure 4).

Overall, the mean RT decreased in the second training session (T_2_) relative to the first (T_1_), and then reduced consistently across the five training sessions, except for a slight increase of the mean RT for the no blur and low blur groups during the fourth training session (T_4_; see Figure 4). The decrease in mean RT in T_5_ relative to T_1_ for no blur, low blur, and severe blur groups were 70.76, 104.26, and 128.58 ms, which represents a significant improvement across the course of the training of 10.8%, *t*(9) = 2.49, *p* = .034; 13.1%, *t*(9) = 4.07, *p* = .003; and 14.7%, *t*(9) = 3.58, *p* = .006, respectively.

#### Measures of improvement: feature search tasks

Mean accuracy was above 94% in all conditions for all tasks and there were no significant differences between conditions (*p* ⩾ .197).

##### Colour search task

**Figure 5.**
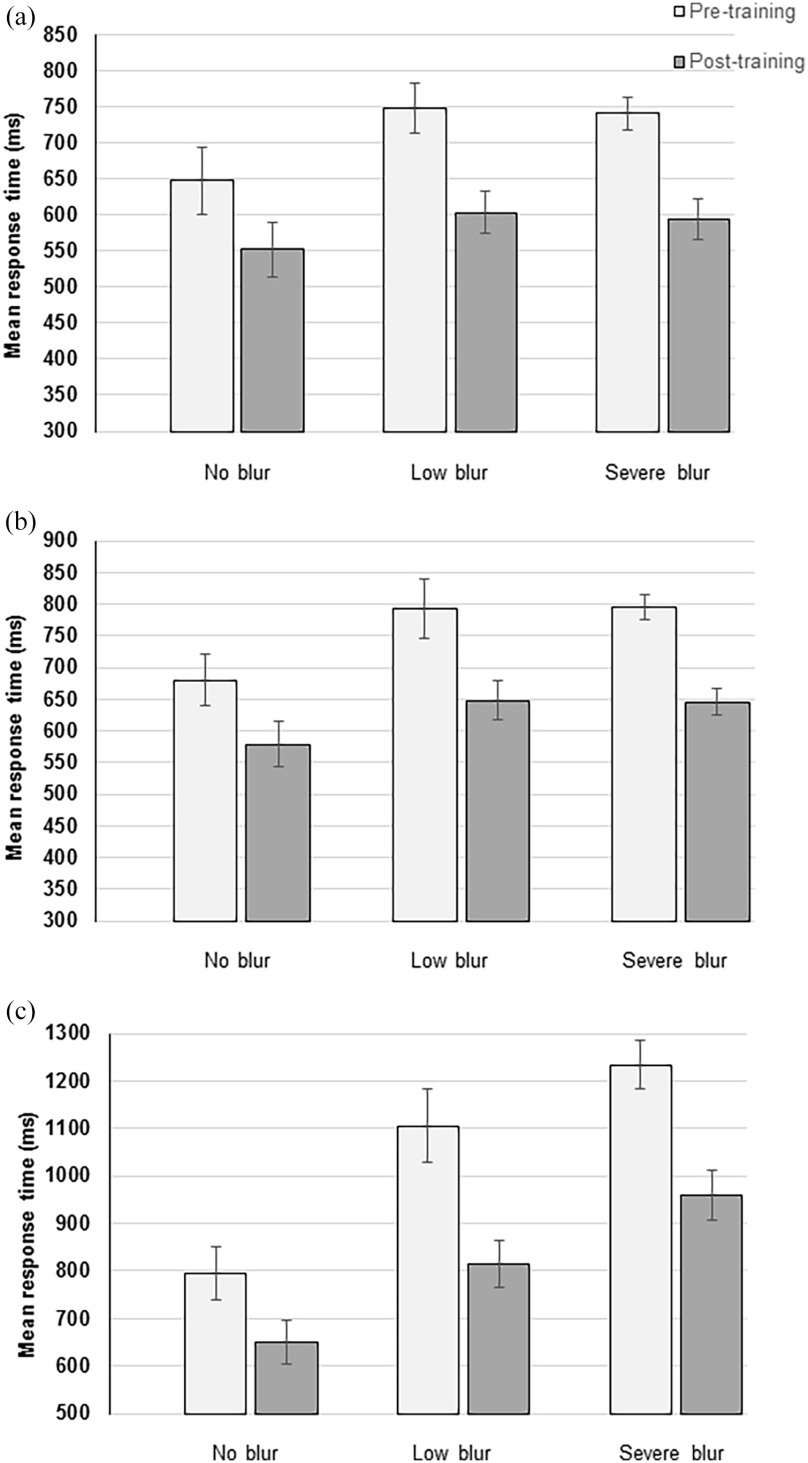
Bar charts to illustrate the mean response time (in milliseconds) across all three Set-sizes for each of the groups pre- and post-training in the (a) colour search task, (b) size search task, and (c) shape search task. The error bars represent the standard error of the mean.

The [3 (Set-size: 4 items, 8 items, and 12 items) × 2 (Session: pre- and post-training)] × 3 (Group: no blur, low blur and severe blur) mixed-model ANOVA on the mean RT revealed a significant effect of Session, *F*(1, 27) = 65.27, *p* < .001; search speed was significantly faster post-training compared with pre-training (see Figure 5a). The remaining main effects and interactions were all non-significant (*p* ⩾ .175), including Set-size; the mean search rate slope was 0.50 ms/item.

##### Size search task

The [3 (Set-size: 4 items, 8 items, and 12 items) × 2 (Session: pre- and post-training)] × 3 (Group: no blur, low blur and severe blur) mixed-model ANOVA on the mean RT revealed significant effects of Session, *F*(1, 27) = 65.17, *p* < .001. As can be seen in Figure 5b, the search speed was significantly faster post-training compared with pre-training. There was also a significant effect of Set-size, *F*(2, 54) = 53.97, *p* < .001; RT was slower as the number of items displayed increased. The mean RT for 4 item displays was 660.47 ms (*SD*: 53.27) and for 12 items it was 719.37 ms (*SD*: 58.82), and the mean search rate slope was 8.78 ms/item. There was no significant effect of Group and interactions were all non-significant (*p* ⩾ .057).

##### Shape search task

The [3 (Set-size: 4 items, 8 items, and 12 items) × 2 (Session: pre- and post-training)] × 3 (Group: no blur, low blur and severe blur) mixed-model ANOVA on the mean RT revealed a significant effect of Session, *F*(1, 27) = 93.24, *p* < .001, with participants faster after training (see Figure 5c). There was also a significant effect of Set-size, *F*(2, 54) = 45.47, *p* < .001; mean RT increased with increasing Set-size from 880.80 ms (*SD*: 89.27) for 4 items, up to 972.47 ms (*SD*: 101.92) for 12 items, and the mean search rate slope was 13.93 ms/item. The main effect of Group was also significant, *F*(2, 27) = 13.20, *p* < .001; mean RT was significantly higher in the low (*p* = .010) and severe (*p* < .001) blur groups compared with the no blur group. The mean RT between the low and severe blur groups was not significantly different (*p* = .228).

**Figure 6.**
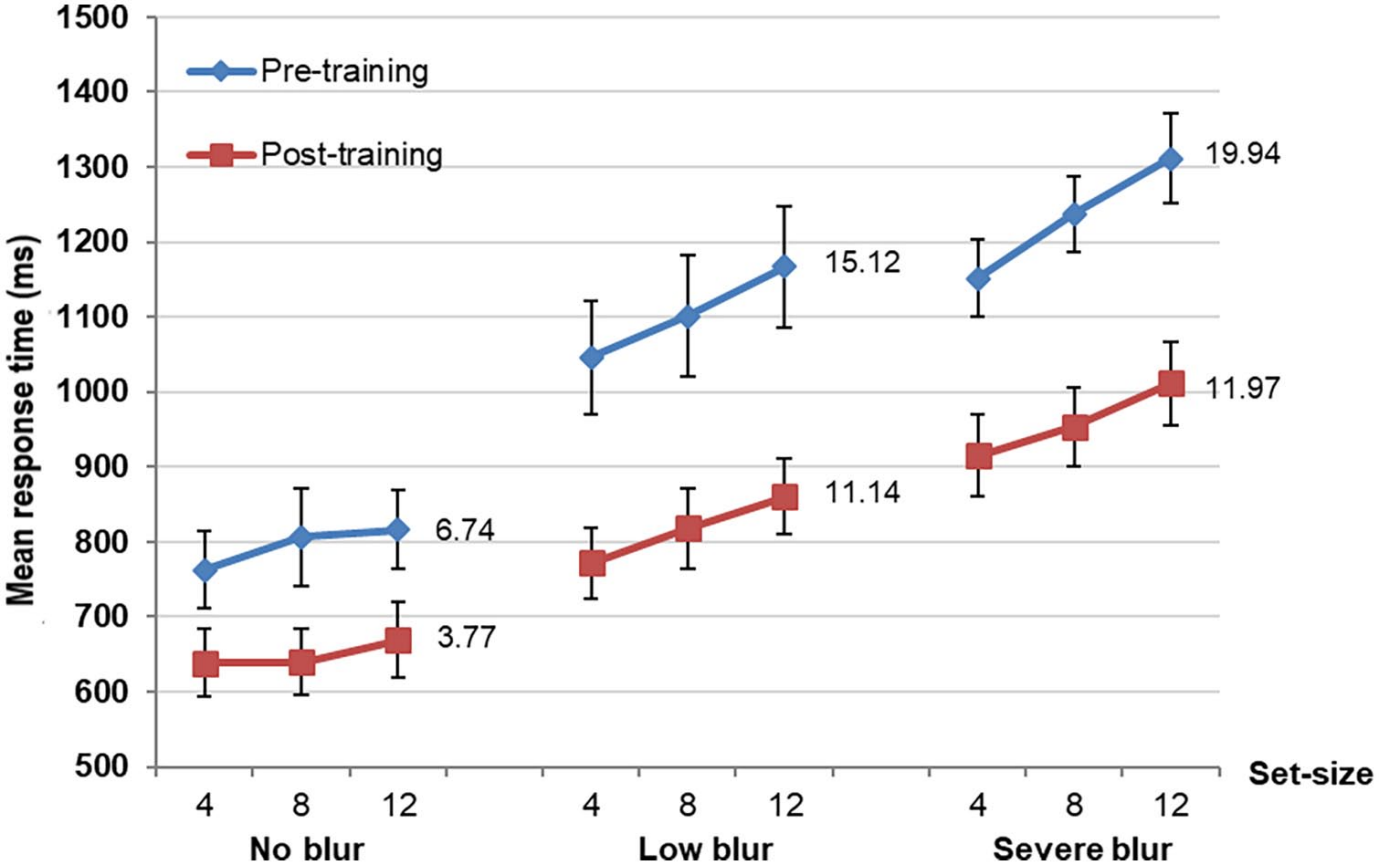
Graph to illustrate the mean search time slopes pre- and post-training across the three different Groups (no blur/low blur/severe blur) in the shape search task. The error bars represent the standard error of the mean, and the mean search slope unit is ms/item.

There were significant interactions between Set-size and Group, *F*(4, 54) = 3.56, *p* = .012, and Session and Group, *F*(2, 27) = 3.43, *p* = .047, but no significant interactions between Set-size and Session, *F*(2, 54) = 2.82, *p* = .068) and Set-size, Session, and Group, *F*(4, 54) = 0.49, *p* = .740, were found. To investigate the significant interaction effects for the shape search task, the mean search slope was calculated for each Session and Group (see Figure 6).

A 2 (Session: pre- and post-training) × 3 (Group: no blur, low blur, and severe blur) mixed-model ANOVA was conducted on the mean search slope data, and revealed significant effects of Session, *F*(1, 27) = 7.04, *p* = .013, and Group, *F*(2, 27) = 5.52, *p* = .010; the mean search slope significantly reduced after the training, and the severe blur group experienced a significantly greater decrease in the search slope relative to the no blur group (*p* = .010). However, there were no interactions between Session and Group, *F*(2, 27) = .663, *p* = .523.

#### Transfer measure: find-the-number search task

The 2 (Session: pre- and post-training) × 3 (Group: no blur, low blur, and severe blur) mixed-model ANOVA on mean RT revealed a main effect of Session, *F*(1, 27) = 6.47, *p* = .017; the mean RT decreased after training (see Table 2). There was also a significant effect of Group, *F*(2, 27) = 34.16, *p* < .001; RT was fastest in the no blur group and slowest in the severe blur group (Table 2). There was no interaction between Session and Group, *F*(2, 27) = 0.77, *p* = .475. The calculated mean improvement in RT was 7.19% (*SD* = 8.8) in the no blur group, 14.62% (*SD* = 18.9) in the low blur group, and 9.75% (*SD* = 24.0) in the severe blur group.

**Table 2.** Table showing the mean RT and mean accuracy for the Find-the-Number task, for both Sessions (pre- and post-training) and for each of the three Groups (no blur, low blur, severe blur).

	Mean RT (ms)		Mean accuracy (%)	
	*Pre-training*	*Post-training*	*Pre-training*	*Post-training*
*No blur*	902.55 (111.08)	838.12 (137.04)	97.30 (1.70)	96.10 (3.03)
*Low blur*	1753.40 (387.75)	1471.61 (370.51)	92.0 (10.06)	97.20 (4.24)
*Severe blur*	2514.09 (673.13)	2243.08 (722.15)	80.70 (14.54)	89.70 (9.70)

RT: response time.

A 2 (Session: pre- and post-training) × 3 (Group: no blur, low blur and severe blur) mixed-model ANOVA was also conducted on mean accuracy data. There was a main effect of Session, *F*(1, 27) = 10.93, *p* = .003; mean accuracy increased after training. There was also a significant effect of Group, *F*(2, 27) = 6.24, *p* = .006; accuracy was highest in the no blur group and lowest in the severe blur group (see Table 2). A significant interaction between Session and Group was also observed, *F*(2, 27) = 5.16, *p* = .013. Post hoc comparisons revealed that accuracy significantly increased after training for the severe blur group (*p* = .008; see Table 2), and remained unchanged in the other groups.

### Discussion

In line with previous research, the results demonstrate that reduced VA can significantly impair visual search performance (Dougherty et al., 2009; Fuhr et al., 2007; Huurneman & Boonstra, 2014; Huurneman et al., 2014; Kuyk et al., 2005; Liu et al., 2007; Satgunam et al., 2012; Senger et al., 2017; Tadin et al., 2012). Specifically, increasing blur from 6/30 (moderate blur) to 6/60 (severe blur) had a marked effect on the speed, but not the accuracy of visual search for size and shape feature tasks. VA did not, however, have a significant effect on the performance of a colour-based feature task. This is the first study to show that the nature of the task can determine the impact that blurring has on search, and that colour is a characteristic that is capable of withstanding reduced VA.

The reason for the poor visual search performance in the shape and size tasks is likely due to reduced salience of visual features that comes with blurring of vision. Earlier studies reported that when the shape information is degraded as a result of blur, a colour cue is more meaningful and helpful in visual search (Markoff, 1972) and object recognition (Wurm et al., 1993). Markoff (1972) conducted a study by blurring black-and-white and colour slides displaying specific targets, like a human or a jeep, that were hidden in real-world backgrounds. For the colour slides, reaction time was shorter compared with the black-and-white slides, and the advantage of colour over black-and-white performance increased with the amount of blur. In the present study, blurring was uniformly distributed throughout the display, affecting both target and distractors equally. As the salience of stimuli gradually reduces, the target which has a different shape appears less distinguishable than its homogeneous, blurred distractors thereby diminishing the pop-out characteristic resulting in more difficult search. Consistent with the information degradation hypothesis, it has been shown that participants in lower VA groups perform worse on tests designed to evaluate executive function, perceptual reasoning, visual search, and processing speed (Bertone et al., 2007; Skeel et al., 2003).

The visual search mechanism underlying the effects of blurring in the size and shape search tasks remains unclear. The predicted normal strategy in feature search tasks is parallel searching (search slope ⩽10 ms/item), whereby the array of items is searched simultaneously and the target easily recognised in a “pop out” manner (Treisman & Gelade, 1980). The colour task was performed in parallel regardless of the amount of blur. Conversely, for the size and shape tasks the search strategy became less efficient (steeper slope) as blur increased. When a search slope exceeds 10 ms/item it is regarded as indicating a serial search strategy, whereby participants examine each item in turn until one item that is perceived as the target is found. As the discriminability between items decreases either through item features or reduced VA, it would seem more serial search strategies are employed to retain accuracy in these tasks. As size and shape are more sensitive to blur, they demonstrated this pattern, while colour tasks were relatively spared by this strategic change.

The results of Experiment 2 showed that irrespective of visual blurring, search performance can significantly improve after training. The mean search improvement obtained in the training itself was actually slightly higher in the blurring groups (14.7% for severe and 13.1% for low) than in the no blurring group (10.8%). It seems likely that this was due to the baseline search speed in the blurring groups being slower relative to the no blurring group. No blur participants, therefore, had less opportunity to gain as much improvement across the course of the training, a phenomenon not uncommon in the literature (see Liu et al., 2007). Examining the performance across all five sessions of training, the mean RT continued to reduce in each session, and the magnitude was greater for the more difficult shape search task. A significant improvement in speed was observed between the first and final sessions regardless of blurring group, and thus it is anticipated that if the training session were to be extended, increased benefits could be gained. Performance did not appear to plateau after the five sessions of training in the severe blurring group, whereas for the no and low blurring group there was minimal change in the final three training sessions. This indicates that there is a chance for greater improvement in the severe blurring group compared with other groups if the training is extended.

As the improvements after training were not only seen in the trained tasks, but also transferred to a non-trained search task (find-the-number) then one possible explanation for the improvements is an enhanced search strategy. Generally, normally sighted participants learn to improve their search speed by initially making several scanning movements, which are then progressively reduced after extensive practice (Ahissar & Hochstein, 1993, 1996; Ellison & Walsh, 1998; Leonards et al., 2002; Sireteanu & Rettenbach, 1995, 2000; Treisman & Gelade, 1980). It is possible that the same modification has been adopted by the optically blurred participants. Although in the present study participants with blurred vision continued to perform the shape task in a serial manner after training, the slope did become shallower indicating that search was becoming more efficient. The current experiment involved only five training sessions. There is little difference in visual search performance between 5 days’ worth of training and 8 days’ worth of training in neurotypical participants with normal or corrected-to-normal vision (Ellison & Walsh, 1998). However, in the case of induced or natural blur it may be that additional training might enable further improvements in search efficiency. Future research could explore whether it is possible for performance to normalise regardless of the extent of blur, the amount of training required for such improvements to be obtained, and importantly recording eye movements would help to explain the mechanism of action.

The experiments reported here involved neurotypical participants with intact visual fields, with induced blurred vision using optical lenses. To make recommendations about how to use visual search for rehabilitation in practice, it is of course important to understand this behaviour in the patient populations of interest. Liu et al. (2007) did report improvements in an easy feature task for patients with severe visual impairment after training, and the present findings suggest that such patients should also still be able to benefit from training using other types of feature search, with transfer to other tasks seen. Future research should explore this possibility, as well as investigating transfer to more complex tasks, including conjunction search, for instance.

In conclusion, it appears that reduced VA impairs performance on various feature visual search tasks, but that participants with blurred vision ranging from mild (6/15) to severe (6/60) can still benefit from visual search training. This means that visual search training could still be an effective rehabilitation tool for those with visual field loss and comorbid blurring.
